# Complete mitochondrial DNA sequence of Norwegian skates (*Raja brachyuran* Lafont, 1871) imported to Korea

**DOI:** 10.1080/23802359.2021.1886003

**Published:** 2021-03-15

**Authors:** Moo-Sang Kim, Jinho Kim, Tae-Wook Kang, Uiseok Jeong, Kang-Rae Kim, In-Chul Bang

**Affiliations:** a theMOAGEN, Daejeon, Republic of Korea; b Department of Life Science & Biotechnology, Soonchunhyang University, Asan, Republic of Korea

**Keywords:** Complete mitochondria genome, Rajiformes, *Raja brachyura*, phylogenetic analysis

## Abstract

In this study, the complete mitochondrial genome of Norwegian skates imported to Korea was sequenced with a circular molecule of 17,121 bp, which consisted of 13 protein-coding genes (PCGs), 2 ribosomal RNAs, 22 transfer RNA genes, and a control region (D-loop). And among these sequences, 193 bp sequence in the D-loop of the genus *Raja* suggested the possibility of being used as a genetic marker for classification of *Raja* and *Dipturus* species. The BI phylogenetic tree by using the nucleotide sequences of 13 PCGs from 15 available mitogenomes of family Rajidae confirmed also that Norwegian skates imported to Korea form a group with *Raja brachyura* species with high branch value, and that this was a species of *Raja brachyura*. As above, these results would be expected to provide for the further understanding on the phylogenetic relationship, taxonomic classification and phylogeography of the family Rajidae.

Skates are cartilaginous fish belonging to the Rajiformes (family Rajidae), and in Korea, they are an important ingredient in popular traditional fermented seafood, however, the quantity of skates (ocellate spot skate, *Okamejei kenojei*; mottled skate, *Beringraja pulchra*) produced in Korea cannot fulfill the demand, thus the imports from countries such as the United States, Norway, New Zealand, Uruguay and Argentina have increased from 103.60 tons in 2018 to 131.72 tons in 2019 (Jang et al. 2017; Choi et al. 2019; Agriculture Forestry Fisheries Information Service 2020). Nevertheless, the information about the country of origin of skates imported to Korea is provided but the species information is unknown. Such information on the origin and scientific name of food will be important information for skates consuming countries in Northeast Asia such as Korea, and for countries that produce them. Therefore, in order to identify the Norwegian skate’s species imported to Korea and develop DNA-markers for interspecific identification from these sequences, we first obtained the complete mitochondrial DNA sequences from this skate.

A sample of Norwegian skates sold in the Korean market was obtained and the genomic DNA was extracted using a Nucleospin Tissue Kit (MACHEREY-NAGEL), and deposited in Soonchunhyang University, Republic of Korea (Accession no. SUC-22000). The genomic library for Next Generation Sequencing (NGS) was constructed using the MGIEasy DNA Library Prep Kit (MGI). After securing NGS raw data by MGISEQ-2000 (MGI), whole mitochondrial DNA sequence of a circular molecule of 17,121 bp was assembled using CLC Assembly cell 5.1.1 (Qiagen), and the final assembled sequence was further annotated using a web-based automatic annotation server, MITOS (Bernt et al. 2013).

NCBI BlastN (Johnson et al. 2008) search of the complete mitochondrial DNA sequence showed a 99.79% similarity to the NC_049865 sequence of the *Raja brachyura* species. Thus, it was presumed that the current sequence belongs to *Raja brachyura* species, and the obtained sequence was registered at NCBI GenBank (Accession no. MT850125).

The mitogenome of *Raja brachyura* included 13 protein-coding genes (PCGs), 22 transfer RNA (*tRNA*) genes, two ribosomal RNA (*rRNA*) genes, and a control region (D-loop) which agrees with a previously reported study (Zhao et al. 2015). Of the thirteen PCGs, twelve PCGs had ATG as a start codon, and the start codons of *cox1* gene was GTG. Four of the PCGs terminated with incomplete stop codons, T (*cox2*, *nd3*, and *nd4*), TA (*nd2*), and the remaining nine ended with complete stop codon (TAA). Twelve PCGs (*nd1*, *nd2*, *cox1*, *cox2*, *atp8*, *atp6*, *cox3*, *nd3*, *nd4L*, *nd4*, *nd5*, *cytb*), fourteen *tRNA*s (*Phe*, *Val*, 2 *Leu*, *Ile*, *Met*, *Trp*, *Asp*, *Lys*, *Gly*, *Arg*, *His*, *Ser*, *Thr*), and two *rRNA*s (12S and 16S *rRNA*) were transcribed on the light strand. The *tRNA* genes were interspersed among the mitochondrial genome and ranged in size from 67 to 75 bp. The *Raja brachyura* mitogenome also contained a small subunit of rRNA (12S *rRNA*) and a large subunit of *rRNA* (16S *rRNA*) as in other fish species (Zhao et al. 2015), which were 967 bp and 1686 bp in length, respectively. Like other fish mitogenomes, these genes were located between the *tRNA^Phe^* and *tRNA^Val^* genes and between *tRNA^Val^* and *tRNA^Leu^* genes, respectively. Finally, a control region (1456 bp) located in between *tRNA^Pro^* and *tRNA^Phe^* was observed.

The sequence of MT850125 identified in this study was 17,121 bp long, whereas the NC_049865 sequence of *Raja brachyura* mitochondrial DNA was 16,925 bp long. The difference of 196 bp between these two sequences was found in D-loop region, it was confirmed that the 193 bp tandem repeat sequence has an additional sequence that repeats twice by using the Tandem Repeats Finder Program (Benson 1999), and it was showed a 97.9% similarity between the two repeat sequences. It was confirmed that similar repeat pattern in the control region of *Raja brachyura* species was seen in GenBank acc. no. AY218363 and AY218362 sequence, and also that the 193 bp sequence has 99.48-82.35% similarity (maximum total score: 351-243) with sequences in control region of these Rajidae from a search limited to *raja* (taxid:7780) and *Dipturus* genus (taxid:117858) for the BlastN organism option. This result suggests that the 193 bp sequence in control region (D-loop) of the genus *Raja* has a potential to be used as a genetic marker for the classification of *Raja* and *Dipturus* species.

The phylogenetic tree was performed within the scope of family Rajidae with 15 available mitogenomes, including *Pristis clavata* (NC_022821) and *Rhinobatos schlegelii* (NC_023951) as outgroup species, using MrBayes v3.2.6 (Huelsenbeck and Ronquist 2001). For the analysis, the nucleotide sequences of 13 protein-coding genes (PCGs) genes were aligned and analyzed using the GTR substitution model and 1,100,000 chain length (Figure 1). The result indicated that the Norwegian skates imported to Korea was grouped together with the *Raja brachyura* (NC_049865) while the ingroup is referred to as three explicitly partitioned lineages in the families Rajidae (Figure 1).

**Figure 1.**
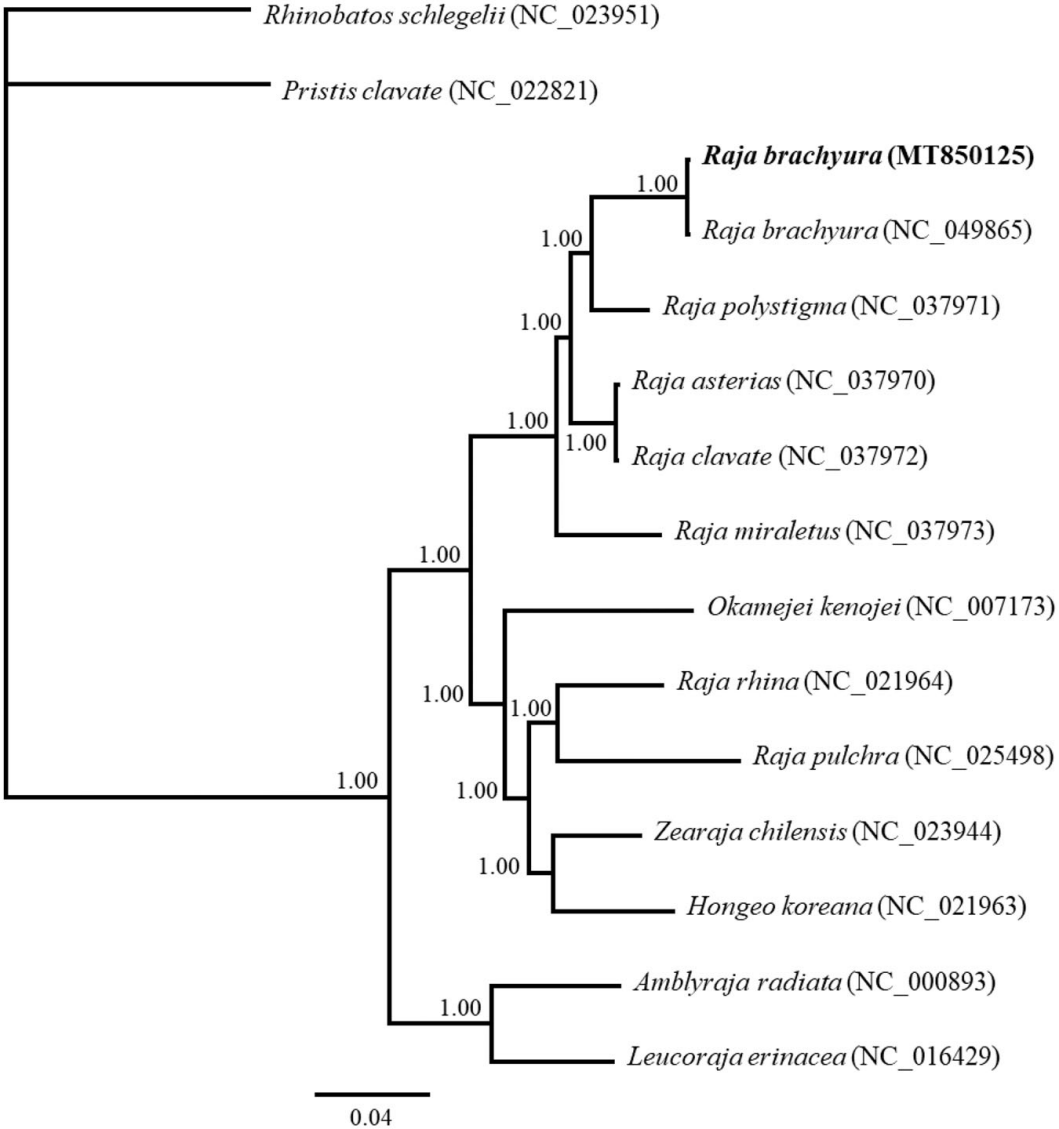
Phylogenetic tree inferred by Bayesian inference based on 13 mitochondrial PCGs from 13 available Rajidae mitogenomes and 2 species as outgroup. GenBank accession numbers of mitogenome sequences used are shown in parentheses. Above the branches were Bayesian posterior probabilities values.

## Data Availability

The genome sequence data that support the findings of this study are openly available in GenBank of NCBI at (https://www.ncbi.nlm.nih.gov/) under the accession no. MT850125. The associated BioProject, SRA, and Bio-Sample numbers are PRJNA690136, SRX9798769 and SAMN17245050, respectively.
